# High phytoremediation and translocation potential of an invasive weed species (*Amaranthus retroflexus*) in Europe in metal-contaminated areas

**DOI:** 10.1007/s10661-023-11422-3

**Published:** 2023-06-01

**Authors:** Bianka Sipos, Dina Bibi, Tibor Magura, Béla Tóthmérész, Edina Simon

**Affiliations:** 1 Department of Ecology, University of Debrecen, Egyetem Square 1, 4032 Debrecen, Hungary; 2 ELKH-DE Anthropocene Ecology Research Group, Egyetem Square 1, 4032 Debrecen, Hungary; 3 MTA-DE Biodiversity and Ecosystem Services Research Group, Egyetem Square 1, 4032 Debrecen, Hungary

**Keywords:** Weeds, Bioaccumulation, Bioindication, Metal contamination, Phytoremediation, Translocation, Roots, Stems, Leaves, Soil

## Abstract

We demonstrated the metal accumulation potential of *Amaranthus retorflexus*, a European weed species, both in moderately and strongly metal-contaminated sites. Metal accumulation in roots, stems, and leaves were studied. We also calculated the bioaccumulation factor (*BAF*), and translocation factor (*TF*) values to quantify the metal accumulation, and translocation between plant organs. Our findings indicated that metal accumulation correlated with metal concentration; that is plant organs accumulated higher concentration of metals in the contaminated area than in the control one. We found that the concentrations of Ba, Mn, Sr and Zn were the highest in leaves, and Al, Cr, Cu, Fe and Pb in roots. High *BAF* value was found for Sr in all studied areas, indicating this metal’s high accumulation potential of *Amaranthus retorflexus*. High *TF* values were found for Al, Ba, Cu, Fe, Mn, Sr and Zn; these metals were successfully transported to aboveground plant organs. We demonstrated that *A. retroflexus*, a fast-growing, rapidly spreading weed in Europe, was especially useful for heavy metal phytoremediation and phytoextraction.

**Supplementary Information:**

The online version contains supplementary material available at 10.1007/s10661-023-11422-3.

## Introduction

Industrialization also increases soil pollution (Khan et al., 2021; Zwolak et al., 2019). Amongst soil contaminants heavy metals is one of the most serious ecological problems all over the world. High concentrations of heavy metals can generate cytotoxic, genotoxic and mutagenic effects in living organisms (Tchounwou et al., 2012). Heavy metals cause significant toxic impact also on plants when their concentration is higher than the threshold limits. Several heavy metals at high concentration are reported to inhibit vegetative growth and decrease the productivity of crops (Ngayila et al., 2009; Rascio & Navari-Izzo, 2011).

Phytoremediation refers to using plants and/or soil microbes to decrease the concentration of contaminants in the soil, water and air (Ali et al., 2013; Mahar et al., 2016). Phytoremediation is a cost-effective, eco-friendly and novel remediation method within bioremediation technologies (Ali et al., 2013). One of the most important advantage of phytoremediation is that it is suitable for application in situ at large field sites where other remediation methods are not cost effective (Vocciante et al., 2017; Li et al., 2018). Phytoremediation involves growing of plants in the contaminated soil using cultivated plants or using the already existing plants for a growth period, to remove contaminants from the site (Dominguez-Rosado & Pichtel, 2004). The usefulness of phytoremediation depends upon duration of exposure, concentration of pollutants, environmental factors and plant characteristics (Anand et al., 2017). During phytoremediation the usage of suitable, fast-growing plant is recommended which has high capacity to accumulate multiple heavy metals in the above-ground parts of the plant. Weed species are preferred for phytoremediation as compared to most hyperaccumulators because of their potential of high biomass and ability to grow in deficient conditions (Ghosh & Singh, 2005).

Earlier studies demonstrated that weeds are good indicator of soil pollution (Salinitro et al., 2019), and they can grow in various soils (Ramírez-Satoyo et al., 2021). Earlier studies reported the usefulness of *Amaranthus retroflexus* in phytoremediation projects. Alsherif et al. (2022) reported high biological concentration factor (BCF), and low translocation factor (*TF*) for Cu, As and Ni of *A. retroflexus*. They discussed stress defence strategies of *A. retroflexus* grown under complex heavy metal-contaminated area. Choudhury et al. (2008) studied the arsenic accumulation potential of *A. retroflexus*. Chehregani et al. (2009) studied the concentration of total Cu, Fe, Zn, Pb and Ni in weed species, and *A. retroflexus* was the only one of them which accumulated these metals in high concentration. Ghazaryan et al. (2021) studied the phytoextraction potential of *Melilotus officinalis* and *A. retroflexus* for Cu and Mo. Their findings indicated that the ability to transport the copper to aboveground parts was more pronounced in *A. retroflexus* than *M. officinalis* (Ghazaryan et al., 2021). Lukatkin et al. (2021) also studied the metal accumulation potential of *A. retroflexus*, which had high accumulation potential in Pb- and Ni-contaminated soils.

Our study aimed to analyse metal accumulation and translocation potential of an early successional pioneer weed, *Amaranthus retroflexus* L., grown in moderately and strongly metal-contaminated soil. Redroot pigweed (*A. retroflexus*) can live on any type of soil and is widely distributed in Europe, so it can be used for bioremediation in a wide range of habitat types. Based on earlier works (Baral et al., 2011; Chunilall et al., 2005) we hypothesized that there is a good phytoremediation potential of this weed species. Moreover, according to the pollution intensity-dependent metal accumulation hypothesis (Tőzsér et al., 2019a) we assumed a positive correlation between the metal accumulation rate and the level of soil contamination.

## Materials and methods

### Study area

**Fig. 1:**
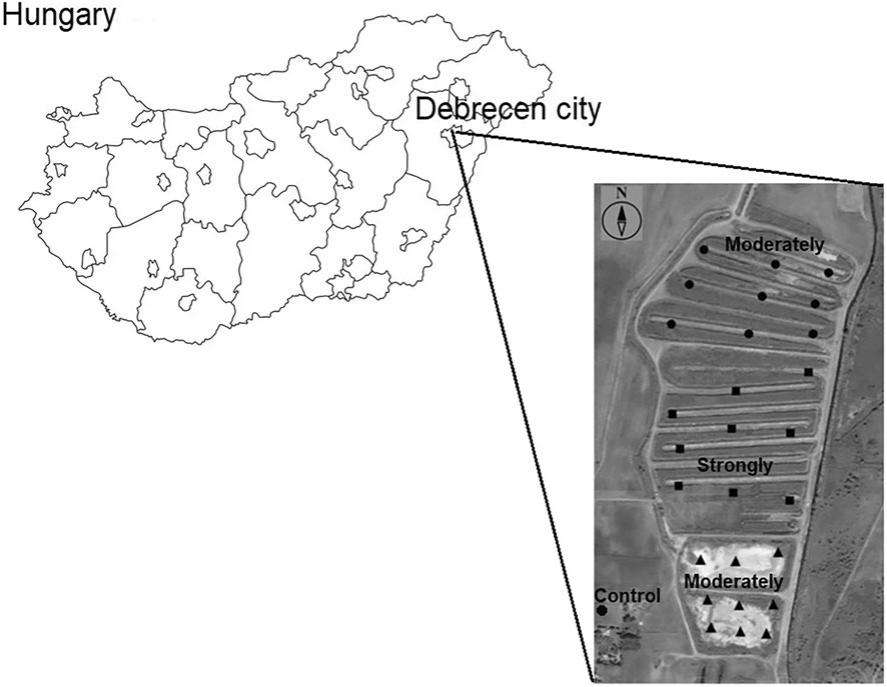
Studied areas with various contamination level

The study area was a recultivated suburban area of Debrecen city, Hungary (47°29′000″N, 21°35′738″E); usually, it is mentioned as Lovász-zug (Fig. 1). The average annual temperature is ± 9.8 °C with − 2.0 °C average monthly temperature in January and ± 22.6 °C in July. The average annual rainfall is 570 mm with 27-mm average monthly precipitation in February and 74 mm in June. The average annual sunshine hours slightly exceed 2000. Between the 1930s and 1950s, the study area functioned as a secondary biological wastewater treatment unit, which was supplemented by physical treatment process later. In the early 2000s, the development of treatment works resulted in the cessation of the lake system, allowing the recultivation of the area to begin. In the initial stage, the sand was spread on the sludge surface in varying thicknesses (80–120 cm) and the two components (sand and sludge) were not mixed. The surface of the area remained slightly uneven, so it is characterized by intermittent water cover, which is increasing from the northern to southern area (Tőzsér et al., 2018, 2019b).

### Studied species

*Amaranthus retroflexus* is native in North America; in Europe it is a weed species (Király, 2009). It is a monoecious plant, which grows to 1.5 m tall, and often infests agronomic fields. The fast-growing species produce more leaf area and preempt growth resources would be expected to be more competitive than slow-growing species. *A. retroflexus* is one of the most frequent weed species in numerous agricultural areas, ranked as the third widespread dicotyledonous weed species in the world (Fried et al., 2020; Kolářová et al., 2023).

### Soil sampling and analysis

Soil samples were collected from three parts of study area (north, middle, south) in October 2020. From each part, five pooled soil samples were collected (*N* = 15). Soil samples were collected with hand spade from 20-cm depth. All samples were put into plastic packages and were stored at ~ 4 °C until laboratory process. The control samples were collected from an uncontaminated area near, but outside the Lovász-zug area. In the laboratory, for the soil moisture content analysis 0.2 g of wet soil samples was put into porcelain pots, with an accuracy of 0.005 g. Then, samples were left overnight at 105 °C. After cooling, the weight of the porcelain pots was measured again. For soil pH measurements, 5 g of soil samples was put into 25-ml plastic centrifuge tubes with an accuracy of 0.005 g. After this, 20 ml of deionized water was added to samples and soil solutions were shaken and left to settle overnight. Soil pH was determined from the solution using a portable multimeter (Hach HQ 40d). For the determination of soil organic matter (SOM), 0.2 (± 0.005) g of soil samples was added into porcelain pots and cremated at 550 °C for 4 h in a muffle furnace (Nabertherm L5/C6, Germany) with loss on ignition method (Balogh et al., 2016; Heiri et al., 2001). For the determination of calcium carbonate content, the samples were cremated at 950 °C in a muffle furnace (Nabertherm L5/C6, Germany) for 2 h. For elemental analysis, 0.2 g of air-dried samples was homogenized using an agate mortar, put into 100-ml glass beakers with an accuracy of 0.005 g, and dried at 105 °C overnight. Soil samples were digested in 10 ml 65% (m/m) HNO_3_ and 200 µl 30% (m/m) H_2_O_2_ on hot plates until total evaporation of the chemicals. Then, samples were diluted with 10 ml 1% (m/m) HNO_3_ into the centrifuge tubes (Simon et al., 2013). The following elements were analysed with inductively coupled plasma optical emission spectrometry (ICP-OES Agilent 5110): Ag, Al, Ba, Bi, Ca, Cd, Co, Cu, Cr, Fe, K, Li, Mg, Mn, Na, Ni, Pb, Sr and Zn. All the concentration values demonstrated in our study refer to dry matter concentrations. Soil (BCR670) CRM was used and the recoveries were within of the 10% of the certified values for the elements.

### Plant sampling and analysis

From each part of the study area samples of *A. retroflexus* were collected in October 2020. From each part of the study area and the control area 10 individuals were randomly collected. All individuals were put into paper bags and were stored in the laboratory to dry. After separating plant parts, roots, stems and leaves were washed (Egwu et al., 2019; Tőzsér et al., 2019b) After air drying for 24 h, samples were dried in a drying oven at 60 °C for 48 h. Root and stem samples were homogenized with laboratory machine, whilst leaf samples were homogenized in an agate mortar and stored in plastic tubes. For elemental analysis, 0.1 g root, stem and leaf samples were digested with 10 ml 65% (m/m) HNO_3_, 2 ml deionized water and 200 µl 30% (m/m) H_2_O_2_. After digestion samples were diluted with 10 ml 1% (m/m) HNO_3_ into centrifuge tubes (Simon et al., 2014, 2021). Elements (Al, Ba, Cd, Co, Cu, Cr, Fe, Mn, Ni, Pb, Sr and Zn) were analysed using inductively coupled plasma optical emission spectrometry (ICP-OES Agilent 5110). Peach leaves (1547) CRM was used and the recoveries were within of the 10% of the certified values for the elements.

### Bioaccumulation and translocation factor

To characterize the degree of uptake, bioaccumulation factor (*BAF*) values were used, based on the soil and plant part concentration values using the following equation:

1 $$BAF=\frac{{C}_{\mathrm{plant\;part}}}{{C}_{\mathrm{soil}}},$$

where *C*_plant part_ is the metal concentration (mg kg^−1^, dry matter) detected in the selected plant part and *C*_soil_ is the metal concentration (mg kg^−1^, dry matter) detected in the growing media (Li et al., 2007; Rezvani & Zaefarian, 2011).

The translocation factor refers to the ratio of metal concentration in selected aboveground plant organs (*C*_aboveground plant part_) and metal concentration in roots (*C*_roots_) (Malik et al., 2010; Mellem et al., 2009):

$$TF=\frac{{C}_{\mathrm{aboveground\;plant\;part}}}{{C}_{\mathrm{roots}}}$$

### Statistical analysis

Element concentrations of plants and soil samples were evaluated by principal component analysis (PCA). Levene test was used to test the homogeneity of variances. For comparing mean concentration values of plants and soil samples one-way ANOVA was used based on studied areas.

## Results

### Metal concentration in soil

**Fig. 2:**
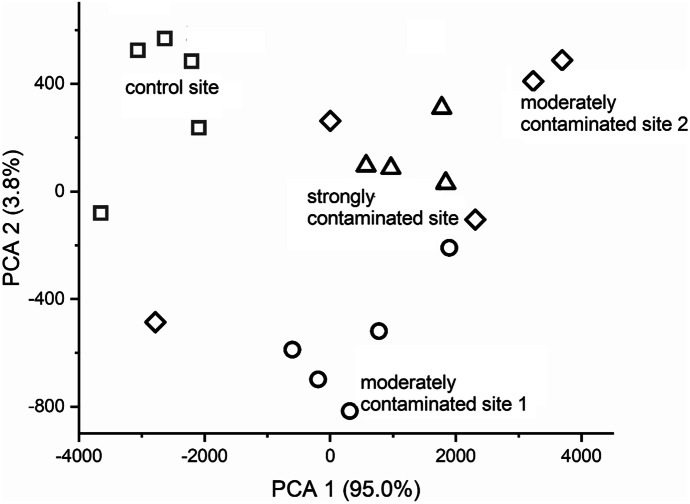
PCA scatterplot based on elemental concentration of soil

We found a significant difference amongst the studied areas based on metal concentration of the soil using PCA analysis (Fig. 2). The concentration of all elements, but Cd, Cu and Zn, was significantly (*p* < 0.05) differed amongst the studied sites (Al: *F* = 3.629, *p* = 0.036; Ba: *F* = 172.844, *p* < 0.001; Cd: *F* = 3.152, *p* = 0.054; Co: *F* = 10.491, *p* < 0.001; Cr: *F* = 393.921, *p* < 0.001; Cu: *F* = 0.988, *p* = 0.423; Fe: *F* = 9.647, *p* = 0.001; Mn: *F* = 4.944, *p* = 0.013; Ni: *F* = 39.887, *p* < 0.001; Pb: *F* = 59.288, *p* < 0.001; Sr: *F* = 51.993, *p* < 0.001, Zn: *F* = 2.084, *p* = 0.143). Concentrations of Ba, Cr and Pb were higher in the contaminated sites than the control one (Table 1).

**Table 1:** Average concentration of metals (± SE) in soil in the studied areas (*N* = 5)

Elements	Control site	Moderately contaminated site 1	Strongly contaminated site	Moderately contaminated site 2	Threshold limit
Al, g/kg	8 ± 1^a^	8 ± 1^a^	9 ± 1^a^	9 ± 1^a^	-
Ba, mg/kg	81 ± 4^a^	147 ± 11^b^	126 ± 8^c^	42 ± 7^d^	250
Cd, mg/kg	1 ± 1^a^	2 ± 1^a^	2 ± 1^a^	1 ± 1^a^	1
Co, mg/kg	4 ± 1^a^	5 ± 1^b^	6 ± 1^bc^	6 ± 1^bd^	30
Cr, mg/kg	12 ± 1^a^	112 ± 10^b^	141 ± 10^b^	17 ± 3^b^	75
Cu, mg/kg	43 ± 3^a^	60 ± 5^b^	57 ± 4^b^	23 ± 4^a^	75
Fe, g/kg	10 ± 1^a^	14 ± 1^b^	14 ± 1^b^	14 ± 2^b^	-
Mn, mg/kg	359 ± 25^a^	385 ± 15^a^	338 ± 7^a^	304 ± 62^a^	-
Ni, mg/kg	13 ± 1^a^	21 ± 1^bc^	25 ± 1^bc^	19 ± 3^b^	40
Pb, mg/kg	25 ± 7^a^	37 ± 3^b^	35 ± 2^b^	8 ± 1^c^	100
Sr, mg/kg	103 ± 9^a^	91 ± 4^b^	84 ± 5^b^	49 ± 9^c^	-
Zn, mg/kg	402 ± 448^a^	216 ± 12^b^	216 ± 17^b^	48 ± 6^c^	100

Different superscript letters indicate significant differences (*p* < 0.05).

### Metal accumulation in plant parts

**Fig. 3:**
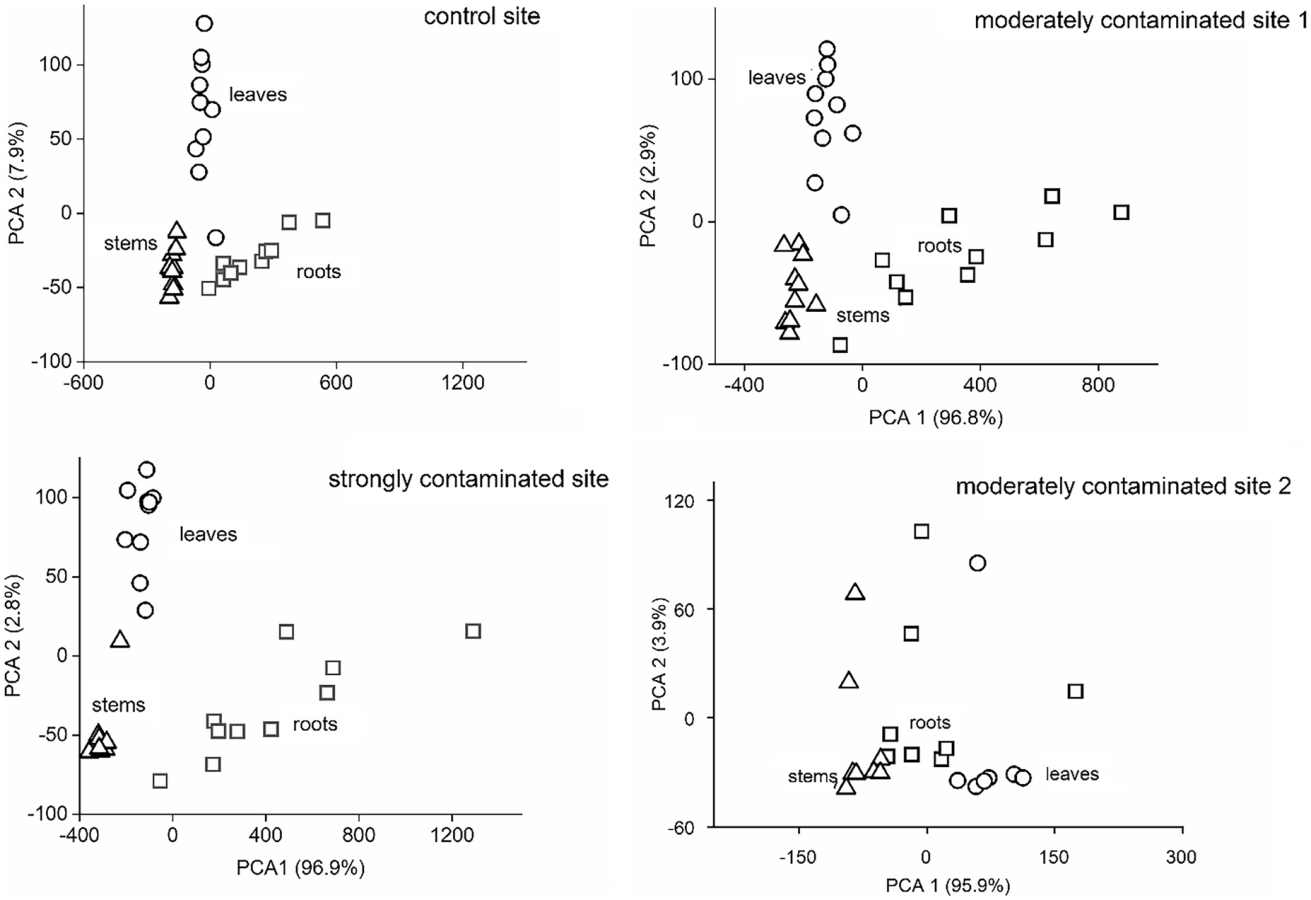
Plot of the principal component analysis (PCA) based on elemental concentration in parts (roots, leaves and stems) of *A. retroflexus* in the studied sites

Based on the metal concentrations, different plant parts were clearly separated from each other in all studied areas (Fig. 3; SM Table 1).

On the moderately contaminated site 1 of the study area, the concentrations of Ba, Mn, Sr and Zn were the highest in leaves, and the concentrations of Al, Cr, Cu, Fe and Pb were the highest in roots. On the strongly contaminated site of the study area, the concentrations of Ba, Mn, Sr and Zn were the highest in leaves, and the concentrations of Al, Cr, Cu, Fe and Pb were the highest in roots. On the moderately contaminated site 2 of the study area concentrations of Ba, Cu, Mn and Sr were the highest in leaves, and concentrations of Al, Cr, Fe, Pb and Sr were the highest in roots (Table 1).

Significant difference was found in Ba, Cr, Cu, Pb and Sr concentrations of *A. retroflexus* roots amongst the studied areas. The highest concentrations of Ba, Cr, Cu, Pb and Sr were detected at the strongly contaminated site (Table 1). The Al, Ba, Cu, Fe, Mn, Sr and Zn concentrations in stems of *A. retroflexus* were also significantly different amongst the studied sites. Concentrations of Ba, Cr, Cu, Pb and Sr were significantly higher in the contaminated sites than in control one (Table 2). The concentrations of Al, Ba, Cr, Cu, Fe, Mn, Sr and Zn in leaves of *A. retroflexus* differed significantly amongst the studied sites. Concentrations of Al, Ba, Cr, Cu, Fe, Mn, Sr and Zn were significantly higher in the contaminated sites, than in the control site (Table 2).

**Table 2:** Average concentration of metals (± SE) in plant parts of *A. retroflexus* in the studied sites

Studied area	Organs	Studied elements, mg kg^−1^ (mean ± standard error)								
		Al	Ba	Cr	Cu	Fe	Mn	Pb	Sr	Zn
Control site	Roots	245 ± 79^a^	11 ± 2^a^	n.d.^a^	4 ± 1^a^	345 ± 132^a^	16 ± 4^a^	2 ± 1^ac^	50 ± 6^a^	24 ± 5^a^
	Leaves	114 ± 17^a^	20 ± 6^a^	0.2 ± 0.1^a^	4 ± 1^a^	166 ± 23^a^	48 ± 12^a^	1 ± 1^a^	147 ± 43^a^	26 ± 1^a^
	Stems	26 ± 7^a^	11 ± 2^a^	n.d.^a^	3 ± 1^a^	46 ± 10^a^	7 ± 2^a^	1 ± 1^a^	66 ± 14^a^	16 ± 3^a^
Moderately contaminated site 1	Roots	221 ± 124^a^	13 ± 5^a^	4 ± 3^b^	5 ± 2^a^	467 ± 390^a^	15 ± 13^a^	3 ± 1^ad^	57 ± 16^a^	29 ± 16^a^
	Leaves	160 ± 43^bc^	47 ± 17^bc^	1 ± 1^b^	4 ± 1^a^	238 ± 66^bc^	20 ± 6^b^	1 ± 1^a^	149 ± 44^a^	57 ± 9^bc^
	Stems	21 ± 8^a^	16 ± 4^bc^	n.d.^a^	2 ± 1^a^	34 ± 11^a^	3 ± 1^b^	1 ± 1^a^	60 ± 10^a^	19 ± 3^a^
Strongly contaminated site	Roots	373 ± 125^a^	16 ± 5^b^	7 ± 3^b^	7 ± 2^b^	686 ± 312^a^	18 ± 7^a^	3 ± 1^ad^	81 ± 21^bc^	35 ± 10^a^
	Leaves	147 ± 27^ac^	52 ± 12^bc^	1 ± 1^b^	4 ± 1^a^	223 ± 31^bc^	21 ± 9^b^	0.3 ± 0.1^b^	219 ± 29^bc^	35 ± 10^ac^
	Stems	43 ± 20^a^	16 ± 5^bc^	n.d.^a^	3 ± 1^a^	77 ± 30^b^	5 ± 2^a^	0.2 ± 0.1^b^	63 ± 10^a^	25 ± 6^b^
Moderately contaminated site 2	Roots	370 ± 167^a^	13 ± 4^a^	1 ± 1^c^	4 ± 1^a^	567 ± 230^a^	18 ± 20^a^	1 ± 1^ac^	66 ± 21^ac^	24 ± 12^a^
	Leaves	110 ± 22^a^	34 ± 13^ac^	n.d.^c^	5 ± 1^a^	194 ± 33^ac^	29 ± 9^b^	n.d.^c^	188 ± 36^ac^	18 ± 3^ab^
	Stems	57 ± 19^b^	14 ± 4^ac^	n.d.^a^	3 ± 1^a^	89 ± 26^b^	8 ± 2^a^	n.d.^c^	78 ± 23^b^	19 ± 5^a^

n.d. indicates that the concentration of the element was below the detection limit (*N* = 10). Different superscript letters indicate significant differences (*p* < 0.05)

### Values of BAF and TF

Higher *BAF* value than 1 was found for the Sr in all studied areas (Table 3). Higher *TF* values than 1 were also found for the Ba, Mn and Sr in all studied areas (Table 4). For the Al and Fe higher *TF* values than 1 were found only in the moderately contaminated site 1 of the area (Table 4). The *TF* value of Zn was higher than 1 in the control site, the moderately contaminated site 1 and the strongly contaminated site. We also found higher value of *TF* than 1 for the Cu but only in the moderately contaminated sites 1 and 2 (Table 4).

**Table 3:** Bioaccumulation factor (*BAF*) (mean ± SE) based on the metal concentration in plant parts and soil

Areas	*BAF*	Elements								
		Al	Ba	Cr	Cu	Fe	Mn	Pb	Sr	Zn
Control site	*BAF*_roots/soil_	0.03 ± 0.01	0.1 ± 0.02	0.01 ± 0.01	0.01 ± 0.01	0.03 ± 0.01	0.1 ± 0.01	0.1 ± 0.1	0.5 ± 0.1	0.1 ± 0.01
	*BAF*_stems/soil_	0.01 ± 0.01	0.3 + 0.1	0.01 ± 0.04	0.01 ± 0.01	0.02 ± 0.01	0.1 ± 0.03	0.03 ± 0.03	**1.4 ± 0.4**	0.01 ± 0.01
	*BAF*_leaves/soil_	< 0.001	0.1 ± 0.03	0.01 ± 0.01	0.01 ± 0.01	< 0.001	0.02 ± 0.01	0.03 ± 0.03	0.6 ± 0.1	0.04 ± 0.01
Moderately contaminated site 1	*BAF*_roots/soil_	0.04 ± 0.03	0.1 ± 0.03	0.03 ± 0.02	0.1 ± 0.03	0.03 ± 0.03	0.04 ± 0.03	0.1 ± 0.03	0.6 ± 0.2	0.1 ± 0.1
	*BAF*_stems/soil_	0.02 ± 0.01	0.3 ± 0.1	0.01 ± 0.01	0.1 ± 0.02	0.02 ± 0.0	0.1 ± 0.01	0.02 ± 0.02	**1.6 ± 0.5**	0.3 ± 0.04
	*BAF*_leaves/soil_	< 0.001	0.11 ± 0.03	0.01 ± 0.01	0.03 ± 0.01	< 0.001	0.01 ± 0.01	0.01 ± 0.01	0.7 ± 0.1	0.1 ± 0.01
Strongly contaminated site	*BAF*_roots/soil_	0.05 ± 0.02	0.1 ± 0.04	0.05 ± 0.02	0.1 ± 0.04	0.05 ± 0.02	0.1 ± 0.02	0.1 ± 0.03	**1.0 ± 0.3**	0.2 ± 0.1
	*BAF*_stems/soil_	0.02 ± 0.01	0.4 ± 0.1	0.01 ± 0.01	0.1 ± 0.01	0.02 ± 0.01	0.1 ± 0.03	0.01 ± 0.02	**2.6 ± 0.4**	0.2 ± 0.1
	*BAF*_leaves/soil_	< 0.001	0.1 ± 0.04	< 0.001	0.1 ± 0.01	0.01 ± 0.01	0.02 ± 0.01	0.01 ± 0.01	**1.3 ± 0.2**	0.1 ± 0.03
Moderately contaminated site 2	*BAF*_roots/soil_	0.04 ± 0.02	0.3 ± 0.1	0.04 ± 0.05	0.2 ± 0.02	0.04 ± 0.02	0.1 ± 0.02	0.1 ± 0.1	**1.4 ± 0.4**	0.5 ± 0.3
	*BAF*_stems/soil_	0.01 ± 0.01	0.8 ± 0.3	< 0.001	0.2 ± 0.03	0.01 ± 0.01	0.1 ± 0.03	0.03 ± 0.07	**3.8 ± 0.7**	0.4 ± 0.1
	*BAF*_leaves/soil_	0.01 ± 0.01	0.3 ± 0.1	< 0.001	0.1 ± 0.02	0.01 ± 0.01	0.03 ± 0.01	< 0.001	**1.6 ± 0.5**	0.4 ± 0.1

*BAF* values higher than 1 marked with bold

**Table 4:** Translocation factor (*TF*) (mean ± SE) based on the metal concentration in plant parts

Elements	Control site		Moderately contaminated site 1		Strongly contaminated site		Moderately contaminated site 2	
	*TF*_stems/roots_	*TF*_leaves/roots_	*TF*_stems/roots_	*TF*_leaves/roots_	*TF*_stems/roots_	*TF*_leaves/roots_	*TF*_stems/roots_	*TF*_leaves/roots_
Al	0.1 ± 0.03	0.5 ± 0.2	0.2 ± 0.2	**1.0 ± 1.0**	0.1 ± 0.1	0.4 ± 0.2	0.2 ± 0.1	0.4 ± 0.2
Ba	**1.0 ± 0.2**	**2.0 ± 0.6**	**1.3 ± 0.6**	**4.1 ± 2.1**	**1.0 ± 0.2**	**3.3 ± 1.0**	**1.1 ± 0.3**	**2.7 ± 0.9**
Cr	< 0.001	< 0.001	< 0.001	< 0.001	< 0.001	< 0.001	< 0.001	< 0.001
Cu	0.6 ± 0.1	0.8 ± 0.1	0.5 ± 0.2	**1.0 ± 0.6**	0.4 ± 0.1	0.6 ± 0.2	0.8 ± 0.1	**1.2 ± 0.2**
Fe	0.1 ± 0.04	0.6 ± 0.2	0.2 ± 0.2	**1.0 ± 1.0**	0.1 ± 0.1	0.4 ± 0.2	0.2 ± 0.1	0.4 ± 0.3
Mn	0.5 ± 0.1	**3.1 ± 1.3**	0.5 ± 0.5	**2.5 ± 2.5**	0.3 ± 0.2	**1.4 ± 1.2**	0.5 ± 0.2	**1.7 ± 0.6**
Pb	0.3 ± 0.4	0.4 ± 0.4	0.1 ± 0.2	0.2 ± 0.3	0.1 ± 0.2	0.1 ± 0.2	< 0.001	< 0.001
Sr	**1.3 ± 0.1**	**3.0 ± 0.7**	**1.1 ± 0.4**	**2.7 ± 0.9**	**1.4 ± 0.3**	**2.9 ± 0.9**	**1.2 ± 0.3**	**3.0 ± 0.6**
Zn	0.7 ± 0.1	**1.1 ± 0.2**	0.8 ± 0.2	**2.3 ± 1.0**	0.8 ± 0.2	**1.1 ± 0.4**	0.9 ± 0.2	0.9 ± 0.3

*TF* values higher than 1 marked with bold

## Discussion

*Amaranthus* species are known as good bioaccumulator plants (Baral et al., 2011; Chunilall et al., 2005; Peter & Gandhi, 2017; Venskutonis & Kraujalis, 2013). We investigated the bioaccumulation potential of *Amaranthus retroflexus* on different contaminated sites. In line with our hypothesis, *A. retroflexus* proved to be a good accumulator species. Furthermore, confirming our hypothesis, there was a positive correlation between its metal accumulation rate and the level of soil contamination. The stems and leaves of *A. retroflexus* accumulated higher concentrations of metals in the contaminated sites than in the control sites. We found that the metal accumulation rate differed significantly amongst plant organs; the concentrations of Ba, Mn, Sr and Zn were the highest in leaves, whilst Al, Cr, Cu, Fe and Pb in roots. So, amongst the plant organs the leaf and root have a prominent role in metal accumulation. Higher *BAF* value than 1 was found only for Sr, indicating high accumulation potential of *A. retorflexus* for this metal.

In our study, Pb concentrations ranged from 1.11 to 3.09 mg/kg in the plant parts of *A. retroflexus*. Phytoextraction studies of other weed species, *Chenopodium album* and *Tripleurospermum inodorum* in the same study area (Tőzsér et al., 2019b), showed that Pb concentration in leaves of *C. album* ranged from 0.03 to 0.06 mg/kg, whilst in stems ranged from 0.07 to 0.08 mg/kg and in roots from 0.06 to 0.1 mg/kg. Furthermore, the Pb concentration in leaves of *T. inodorum* was between 0.03 and 0.06 mg/kg, in stems 0.03 and 0.05 mg/kg and in roots 0.06 and 0.16 mg/kg (Tőzsér et al., 2019b). Both weeds showed lower Pb accumulation rate than *A. retroflexus*. Also, lower Pb concentration (between 0.0014 and 0.0018 mg/kg) was found in *A. retroflexus* along the Makera Drain in Nigeria during phytoextraction on irrigated areas (Mohammed & Folorunsho, 2015). In contrast, in a study with *Amaranthus viridis* in Lagos, Nigeria, lead concentrations were 68–152 mg/kg in leaves, 48–131 mg/kg in stems and 50–369 mg/kg in roots. The study area, however, was located along a highway with extreme pollution (Atayese et al., 2008). Also in Lagos, Nigeria Adewuyi et al. (2010) investigated metal uptake of *Amaranthus caudatus* at a landfill when Pb concentrations in the plant ranged from 68.7 to 145 (mg/kg). The highest Pb concentration in the *Amaranthus tricolor* was 132.69 mg/kg in EDTA-treated soil of a sewage-contaminated area in El-Gabal El-Asfar in Egypt (Awad et al., 2021).

In our study, the Zn concentration in the leaves of *A. retroflexus* ranged from 17.84 to 57.02 mg/kg, stems from 18.65 to 25.42 mg/kg, respectively, and in roots ranged from 23.49 to 34.58 mg/kg. In the same area in the leaves of *C. alba* the Zn concentration ranged 2.77–5.92 mg/kg, 1.08–1.46 mg/kg in stems and 1.29–1.71 mg/kg in roots. Whilst in the leaves of *T. inodorum* the Zn concentration was 1.68–2.92 mg/kg, 1.42–2.70 mg/kg in the stem and 1.36–2.89 mg/kg in the root (Tőzsér et al., 2019b). The zinc uptake of *A. caudatus* was also analysed in a landfill of Lagos, Nigeria. The concentration of Zn in the plant ranged from 14.6 to 39.9 mg/kg (Adewuyi et al., 2010) which was like our finding. *A. caudatus* was also studied in Ibadan, Nigeria to monitor phytoextraction capacity. The measured values were higher in stems (105.75 mg/kg) and in roots (92.15 mg/kg than in our plant organs (Thomas & Omueti, 2012). Zn uptake by *A. retroflexus* was also investigated along irrigated areas of Mohammed and Folorunsho’s (2015) studies (Makera Drain, Nigeria) where the Zn concentration ranged from 14.19 to 69.07 mg/kg. Zn uptake of *Amaranthus hybridus* L. was also studied in Nigeria where the Zn concentration in leaves ranged from 14.2 to 37.2 mg/kg, in stems from 12.7 to 21.4 mg/kg and in roots ranged from 11.2 to 29 mg/kg (Oluwatosin et al., 2009). Chinmayee et al. (2012) also studied the zinc uptake of *Amaranthus spinosus* in soils treated with zinc sulphate under laboratory conditions. Zn accumulation was between 78 and 364 mg/kg in the leaves, 64 and 348 mg/kg in the stems and 82 and 234 mg/kg in the roots (Chinmayee et al., 2012) which are lower concentration values compared to our results. In Egypt, Awad et al. (2021) tested the *A. tricolor*’s bioaccumulation potential in soil treated with ethylene diamine tetraacetic acid (EDTA), poultry litter extract (PLE), vinasse sugarcane (VSC) and humic acid (HA). The highest Zn concentration was 185.64 mg/kg in the plants that grew in the soil with added EDTA (Awad et al., 2021).

In our study the copper concentration in the leaves of *A. retroflexus* ranged from 4.20 to 4.97 mg/kg, in stems from 1.20 to 3.18 mg/kg and in roots ranged from 4.30 to 7.30 mg/kg. In the same study area, the concentration of Cu in leaves of *C. album* ranged from 0.55 to 12.2 mg/kg, in stems from 0.31 to 1.98 mg/kg and in roots ranged from 0.37 to 0.44 mg/kg. The copper concentrations of *T. inodorum* in leaves ranged from 0.65 to 22.2 mg/kg, in stems ranged from 0.27 to 0.33 mg/kg and in roots ranged from 0.54 to 0.64 mg/kg (Tőzsér et al., 2019b). Mohammed and Folorunsho (2015) also studied the concentration of Cu in *A. retroflexus* in irrigated areas, but they found higher values compared to our results (14.19–69.07 mg/kg) (Mohammed & Folorunsho, 2015). Concentration of copper was between 171 mg/kg and 209 mg/kg in *A. caudatus* in a Lagos landfill (Adewuyi et al., 2010). Whilst in *A. spinosus* the concentration in plants grown on soil contaminated with copper sulphate was 50–80 mg/kg in leaves, 23–133 mg/kg in stems and 93–143 mg/kg in roots (Chinmayee et al., 2012). In a study, the highest Cu concentration in *A. tricolor* was 36.54 mg/kg, where EDTA was added to the soil (Awad et al., 2021).

In our study the concentration of Fe in *A. retroflexus* leaves ranged from 193.81 to 238.40 mg/kg, in stems from 33.66 to 89.98 mg/kg and in roots ranged from 466.98 to 686.46 mg/kg. In the same study area, the concentration of Fe in leaves of *C. album* ranged from 6.52 to 29.6 mg/kg, in stems from 1.99 to 5.44 mg/kg and in roots ranged from 4.92 to 15 mg/kg. The concentrations of Fe in *T. inodorum* leaves ranged from 9.23 to 60.4 mg/kg, in stems from 05.84 to 7.14 mg/kg in roots ranged from 17.3 to 32.6 mg/kg (Tőzsér et al., 2019b). Mohammed and Folorunsho (2015) and Adewuyi et al. (2010) also studied the Fe uptake in *A. retroflexus* and *A. caudatus*. In these studies, the concentration of Fe in plants ranged from 6.75 to 21.8 mg/kg.

In our study Cr was detected at low levels in some organs (roots, stems and leaves) of *A. retroflexus*. In a study conducted in the same study area, *C. album* also accumulated Cr at a low rate, as Cr concentration in the leaf was 0.06 mg/kg, in the stem 0.03 mg/kg and in the root 0.13 mg/kg. In *T. inodorum* the measured concentrations of chromium in leaves, stems and roots were 0.09, 0.15 and 0.37 mg/kg, respectively. Cr was also detected at low concentration (0.058–2.80 mg/kg) in *A. retroflexus* at the area of Lagos (Mohammed & Folorunsho, 2015). Whilst Chinmayee et al. (2012) measured high Cr concentration in *A. spinosus L*. in leaf (22–30 mg/kg), in stem (15–22 mg/kg) and in root (18–28 mg/kg).

In our study the concentration of Mn in the leaves of *A. retroflexus* ranged from 20.20 to 29.01 mg/kg, in the stems from 3.47 to 7.91 mg/kg and in the roots ranged from 43 to 18.32 mg/kg. In a previous study on the same area, Mn concentration was 1.29–4.94 mg/kg in the leaves of *C. album*, 0.47–0.90 mg/kg in the stems and 0.51–1.31 mg/kg in the roots. In *T. inodorum* the measured concentration of Mn was 3.55–9.56 mg/kg in the leaves, 1.44–3.77 mg/kg in the stems and 1.47–3.09 mg/kg in the roots (Tőzsér et al., 2019b). In a Lagos landfill Mn concentration in *A. caudatus* ranged from 2.52 to 11.2 mg/kg (Adewuyi et al., 2010).

## Conclusion

*Amaranthus retroflexus* is fast-growing, rapidly spreading weed species in Europe. We demonstrated that *A. retroflexus* have a good metal accumulation and translocation potential, especially for Al, Ba, Cu, Fe, Mn, Sr and Zn. *A. retroflexus* is widely distributed in metal-contaminated soils suggesting a promising opportunity for metal phytoremediation, especially for phytoextraction.

## Supplementary Information

Below is the link to the electronic supplementary material.

Supplementary file1 (DOCX 13 KB)

## Data Availability

Not applicable.
