# Supplementary material for: High phytoremediation and translocation potential of an invasive weed species (Amaranthus retroflexus) in Europe in metal-contaminated areas
Source: Environ Monit Assess. 2023 Jun 1;195(6):790. doi: 10.1007/s10661-023-11422-3 (PMC10234916; doi:10.1007/s10661-023-11422-3)
Supplement: Supplementary file 1 — Supplementary file1 (DOCX 13 KB) [file 10661_2023_11422_MOESM1_ESM.docx]

Supplementary Materials

Supplementary Table 1. Results of the One-way ANOVA based on the elemental concentration of *A. retroflexus* in the studied areas.

| Elements | Factors | F | p value |
| --- | --- | --- | --- |
| Al | Site | 2.876 | 0.040 |
|  | Organ | 81.983 | <0.001 |
|  | Site* Organ | 2.018 | 0.069 |
| Ba | Site | 19.105 | <0.001 |
|  | Organ | 136.582 | <0.001 |
|  | Site* Organ | 8.211 | <0.001 |
| Cr | Site | 31.059 | <0.001 |
|  | Organ | 55.429 | <0.001 |
|  | Site* Organ | 17.258 | <0.001 |
| Cu | Site | 10.248 | <0.001 |
|  | Organ | 73.376 | <0.001 |
|  | Site* Organ | 8.156 | <0.001 |
| Fe | Site | 3.906 | 0.011 |
|  | Organ | 76.624 | <0.001 |
|  | Site* Organ | 2.093 | 0.060 |
| Mn | Site | 12.513 | <0.001 |
|  | Organ | 104.799 | <0.001 |
|  | Site* Organ | 9.416 | <0.001 |
| Pb | Site | 5.368 | 0.002 |
|  | Organ | 59.668 | <0.001 |
|  | Site* Organ | 2.205 | 0.048 |
| Sr | Site | 22.321 | <0.001 |
|  | Organ | 216.422 | <0.001 |
|  | Site* Organ | 1.871 | 0.092 |
| Zn | Site | 23.509 | <0.001 |
|  | Organ | 30.069 | <0.001 |
|  | Site* Organ | 12.391 | <0.001 |
